# Copy numbers of mitochondrial genes change during melon leaf development and are lower than the numbers of mitochondria

**DOI:** 10.1038/s41438-019-0177-8

**Published:** 2019-08-11

**Authors:** Jia Shen, Yuejian Zhang, Michael J. Havey, Weisong Shou

**Affiliations:** 1 Institute of Vegetables, Zhejiang Academy of Agricultural Sciences, 310021 Hangzhou, China; 2 USDA-ARS and Department of Horticulture, University of Wisconsin, Madison, WI 53706 USA

**Keywords:** Plant cell biology, Plant genetics

## Abstract

Melon is a useful plant species for studying mitochondrial genetics because it contains one of the largest and structurally diverse mitochondrial genomes among all plant species and undergoes paternal transmission of mitochondria. We used droplet digital (dd) PCR in combination with flow cytometric determination of nuclear DNA quantities to determine the absolute per-cell copy numbers of four mitochondrial genes (*nad9*, *rps1*, *matR*, and *atp6*) across four stages of melon leaf development. The copy numbers of these mitochondrial genes not only varied during leaf development but also differed among each other, and there was no correlation between the copy numbers of the mitochondrial genes and their transcript levels. The gene copy numbers varied from approximately 36.8 ± 4.5 (*atp6* copies in the 15th leaf) to approximately 82.9 ± 5.7 (*nad9* copies in the 9th leaf), while the mean number of mitochondria was approximately 416.6 ± 182.7 in the 15th leaf and 459.1 ± 228.2 in the 9th leaf. These observations indicate that the leaf cells of melon do not contain sufficient copies of mitochondrial genes to ensure that every mitochondrion possesses the entire mitochondrial genome. Given this cytological evidence, our results indicate that mtDNA in melon exists as a sub-genomic molecule rather than as a single-master circle and that the copy numbers of individual mitochondrial genes may vary greatly. An improved understanding of the molecular mechanism(s) controlling the relative prevalence and transmission of sub-genomic mtDNA molecules should provide insights into the continuity of the mitochondrial genome across generations.

## Introduction

Mitochondria are vital organelles critical for energy production, metabolism, and cell homeostasis. The mitochondrial DNA (mtDNA) of plants varies greatly in size—from 208 kb to 11.3 Mb^1,2^; however, a relatively large plant mtDNA does not disproportionately encode relatively more genes^3,4^. According to classic models, mtDNA exists as a circular chromosome that retains its ancestral bacterial-like architecture^5^. Unlike nuclear DNA in diploids, which have two copies of each gene per cell, multiple copies of mtDNA are present in every mitochondrion, and there are multiple mitochondria in every plant cell. Accordingly, it is widely held that each cell possesses numerous full copies of mtDNA, and this concept of mtDNA copy number is widely assumed in studies of mitochondrial function and transmission^6–8^. However, recent studies on the structure of mtDNA in *Arabidopsis* and tobacco have shown that mtDNA can be divided into sub-genomes and that mitochondrial genes are distributed across different sub-genomic molecules^9,10^. This complex structure of plant mtDNA raises questions about spatial or temporal differences in mtDNA copy numbers. Previous research has revealed the existence of a strong regulatory system to maintain mtDNA copy numbers during reproduction^6,11,12^; however, little is understood about the changes in mtDNA levels in plant cells during vegetative growth.

Melon (*Cucumis melo* L.) is a unique angiosperm because it shows paternal mitochondrial transmission^13^ and it has one of the largest mtDNA molecules at 2740 kb^14,15^. Sequencing of the melon mtDNA produced five scaffolds and four unscaffolded contigs^15^. The genome contains a high number of repetitive sequences, which contribute to the expansion of the melon mtDNA and allow recombination to produce structurally rearranged molecules^15,16^. Therefore, melon is an excellent system for studying mtDNA dynamics during vegetative and reproductive growth. Here, we studied the copy numbers of mitochondrial genes localized on different scaffolds to estimate absolute copy numbers during melon leaf development. Our results revealed that the copy numbers of mitochondrial genes differed from each other and changed during melon leaf development, and that the mtDNA of melon is divided among small sub-genomic molecules distributed across mitochondria.

## Results

### Sampling of leaves during melon development

To encompass the stages of melon leaf development for ploidy determination and DNA analyses, leaves were harvested when the 9th leaf was fully expanded and the 12th, 15th, and 18th leaves were at different growth stages. During this process, the cells of leaves are believed to be dividing and expanding. Therefore, the 9th (oldest), 12th, 15th, and 18th (youngest) leaves from the melon double haploid DHL92 were harvested at the same time. The mean sizes of leaf cells were measured by paraffin sectioning, and the width of cells increased gradually during leaf development (Table 1). In contrast, the lengths of cells increased dramatically from the 12th to the 9th leaf, and there was no significant change from the 18th to the 12th leaf. These results reflect the importance of cell division in the early stages and cell expansion in the late stages of leaf development.

**Table 1:** Size of the leaves and cells at four different stages of leaf development

Leaf	Leaf		Cell	
	Length (cm)	Width (cm)	Length (μm)	Width (μm)
The 18th	2.36 ± 0.16	2.75 ± 0.24	12.94 ± 1.42	7.56 ± 0.90
The 15th	4.76 ± 0.37	5.11 ± 0.45	12.4.3 ± 1.50	14.11 ± 2.35
The 12th	9.20 ± 0.55	11.33 ± 0.60	12.71 ± 2.79	20.19 ± 1.80
The 9th	12.80 ± 0.61	16.36 ± 0.83	24.90 ± 4.44	24.50 ± 4.46

### Mean nuclear C levels during leaf development

**Fig. 1:**
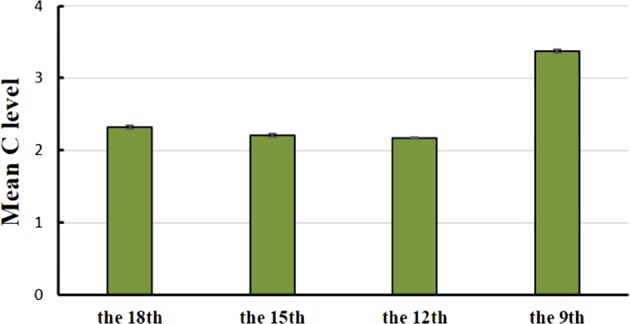
Mean C level of nuclei at the four stages of melon leaf development

The mean nuclear DNA content of leaf cells was highly variable depending on developmental stage, likely due to endoreduplication^17,18^. For this reason, we used flow cytometry to measure the ploidy level of the nuclei before quantifying the copy numbers of mitochondrial genes relative to those of the nuclear DNA during leaf development. We found evidence for endopolyploidization during melon leaf development. Although the diploid (2C) nuclei represented the most common ploidy level, tetraploid (4C), octoploid (8C), and hexadecaploid (16C) nuclei were found. It is possible that parts of cells in the leaves might be in the G2 phase of the cell cycle and therefore temporarily tetraploid, but observations of higher ploidy levels (up to 16C) indicated that endopolyploidization had occurred in the nuclei of melon leaves, and therefore, the tetraploidy values may truly be due to endopolyploidization. Similar ploidies were found at the other developmental stages; the mean C level sharply increased in the 9th leaf but then decreased in the 15th and 12th leaves (Fig. 1). The mean C levels of the four stages of leaf development were 2.32 ± 0.02, 2.21 ± 0.02, 2.18 ± 0.01, and 3.37 ± 0.03, respectively. Overall, the highest mean C level was found in mature (9th) leaves, which indicated that endopolyploidization had occurred in the mature leaves. A similar phenomenon was reported for other plant species, including *Arabidopsis*^19^ and tomato^20^. These findings imply that the increase in endopolyploidization coupled with tissue differentiation is indicative of the degree of cell growth and development. In a study of the relationship between cell size and endoreduplication in the flowers of orchids, Lee et al. suggested that endoreduplication was a contributing factor to cell growth^21^. Based on our data about the size of leaves and cells (Table 1), we observed a dramatic increase in cell size during leaf maturity, which may support their hypothesis.

### Estimated copy numbers of mitochondrial genes in leaves

**Fig. 2:**
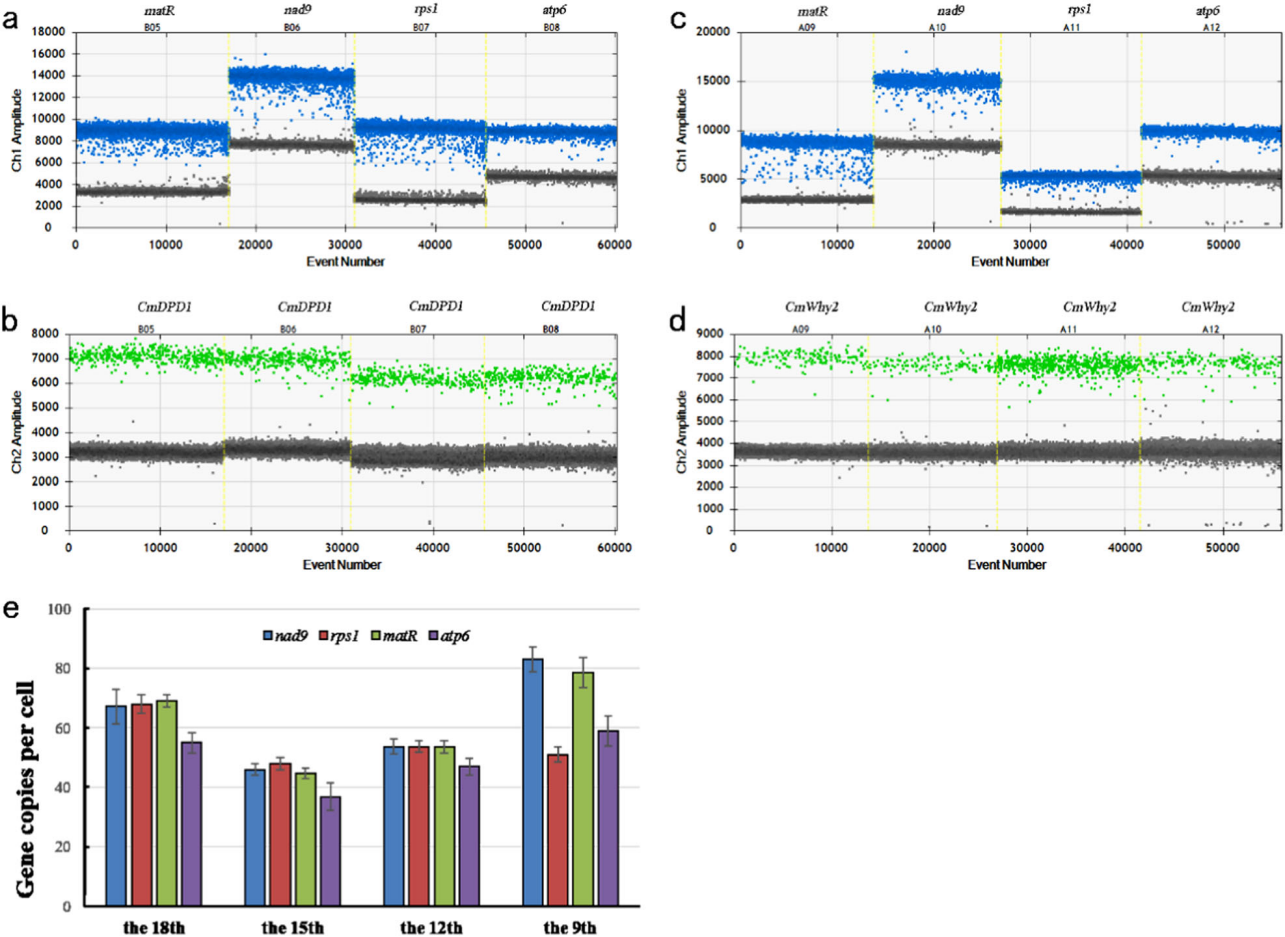
Copy numbers of mitochondrial genes per cell at the four stages of melon leaf development. **a** and **c**, Clusters showing the droplet distribution after amplification of melon leaf total DNA templates at the 18th-leaf stage with specific primers and probes; intensity collected via QX200 filters suited for FAM (Ch1). Left to right: clusters showing the droplet distribution after amplification with specific probes for detecting the mitochondrial genes *matR*, *nad9*, *rps1*, and *atp6*, respectively. **b** and **d**, Clusters showing the droplet distribution after amplification of melon leaf total DNA templates at the 18th-leaf stage with the specific primers and probes; intensity collected via QX200 filters suited for HEX (Ch2). Left to right: clusters showing droplet distribution after amplification with probes for detecting the nuclear gene *CmDPD1*. The mitochondrial and nuclear genes detected by hydrolysis probes with different fluorophores occurred in the same reaction. **e**, The absolute copy number of four mitochondrial genes (*nad9*, *rps1*, *matR*, and *atp6*); the nuclear-encoded single-copy genes *CmDPD1* and *CmWhy2* were used as internal standards. The per-cell copy numbers of mitochondrial genes were calculated with the absolute copy number and the mean C levels

To measure the copy numbers of mitochondrial genes at the four developmental stages of the leaves, total DNA was extracted from leaves that were the same age as those used for the determination of nuclear ploidy levels. Four mitochondrial genes (*nad9*, *rps1*, *matR*, and *atp6)* were chosen as targets for ddPCR analyses. These genes are imperative for the critical processes of ATP synthesis, electron transport, and gene expression, which are core functions of mitochondria. These four genes are distributed across the physical map of the mitochondrial genome of melon^15^. *Nad9*, *rps1*, and *matR* are located on the largest scaffold (Scaffold 1_2), and *nad9* and *rps1* are separated by an intergenic region of only 144 bp, and *atp6* is on scaffold 3. Two single-copy, nuclear-encoded (*CmDPD1* and *CmWhy2*) genes were used as internal standards^7,11,12^. Accordingly, the copy numbers of the nuclear genes per cell estimated the ploidy levels, and the copy number of mitochondrial genes per cell was calculated on the basis of the absolute copy number in units of the mitochondrial and nuclear genes and the mean C value. To eliminate potential errors introduced in the experimental operation, multiplex ddPCR simultaneously measured the absolute copy number of the mitochondrial and nuclear genes by hydrolysis probes with different fluorophores in the same reaction (Fig. 2a, b). To evaluate the effects of the two endogenous reference genes, ddPCR was also performed to measure the absolute copy number of *CmDPD1* and *CmWhy2* by hydrolysis probes with different fluorophores in the same reaction. The results showed impressive consistency across the four stages (Supplementary Table S2), although their positive microdroplets did not coincide completely. This finding may be due to genome breakage during DNA extraction or due to the generation of droplets.

The copy numbers of mitochondrial genes initially decreased but then increased, peaking in the mature leaves (Fig. 2c), except for *rps1*, whose copy numbers did not increase in mature leaves. Compared with the levels of chloroplast genes in plant cells (>1000 copies)^18^, the copy number of mitochondrial genes was much lower in all samples. The highest copy number was found in mature (9th) leaves, with approximately 82.9 copies of *nad9*. The lowest copy number was in the 15th leaf, with approximately 36.8 copies of *atp6*. However, the copy numbers of individual mitochondrial genes were not identical throughout leaf development (Fig. 2c), and the copy numbers of *atp6* were much lower than those of the other three genes (*nad9*, *rps1*, and *matR*). There were no significant differences among copy numbers of *nad9*, *rps1*, and *matR* in the 18th, 15th, and 12th leaves. These results suggest that the mitochondrial genes of melon are not carried on a single-master circular chromosome and instead are distributed across sub-genomic molecules at varying frequencies. Therefore, the copy numbers of individual mitochondrial genes may be independently controlled.

### Transcript levels of mitochondrial genes

**Fig. 3:**
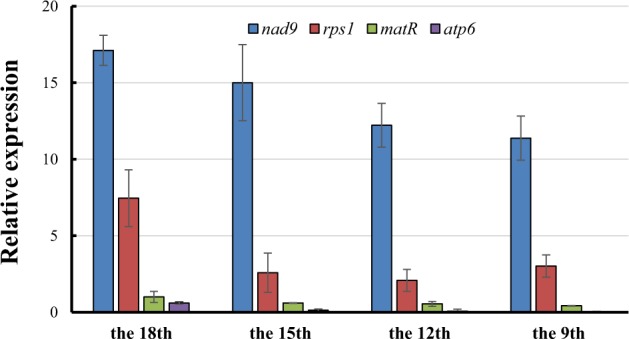
Transcript levels of mitochondrial genes at the four stages of melon leaf development. The relative amounts of transcripts of four mitochondrial genes, *nad9*, *rps1*, *matR*, and *atp6*, were measured via quantitative real-time RT-PCR. The relative transcript levels of the nuclear-encoded housekeeping gene *Actin* were used to normalize the qPCR output

The transcript levels of mitochondrial genes at four stages of leaf development were measured by quantitative RT-PCR. The four mitochondrial genes analyzed do not have introns^15^, and the same primers used in ddPCR were also used in qRT-PCR. Nuclear-encoded *Actin* was used as the control. The expression of all four mitochondrial genes showed a tendency to decrease during leaf development (Fig. 3), and the highest levels were observed in the youngest (18th) leaves. At all four developmental stages, the transcript levels of the four mitochondrial genes were significantly different. The order of the transcript expression levels was *nad9* > *rps1* > *matR* > *atp6*, which did not change throughout the four developmental stages. The transcript levels of *atp6* were undetectable in the 9th-stage leaves. As before, the transcript levels of mitochondrial genes showed no correlation with the mitochondrial gene copy numbers.

### Numbers of mitochondria per cell

**Fig. 4:**
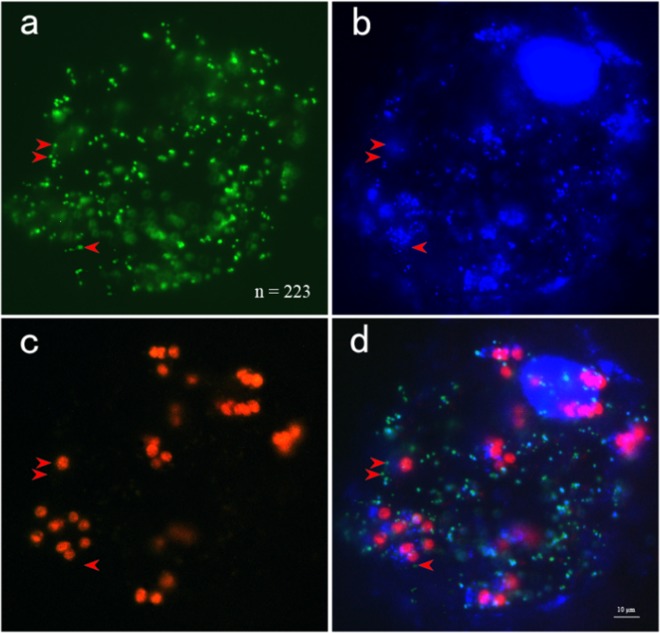
Stained protoplasts isolated from melon. Leaves of melon transformed with a mitochondrially targeted GFP (35S-coxIV-GFP) were stained with DAPI and visualized at excitation wavelengths for green fluorescent protein (**a**) and DAPI (**b**). The number of mitochondria was 223, as determined by ImageJ software. Red autofluorescence was observed from the chloroplasts (**c**). It is clear that mtDNA signals were not present in a portion of the mitochondria after image merging (**d**). The red arrows highlight mitochondria without mtDNA signals. Bar = 10 μm

The numbers of mitochondria per cell were determined by microscopy after DAPI staining of protoplasts from the four stages of melon leaf development. Mitochondria within each protoplast were counted for 30 randomly chosen protoplasts from three independent isolations at each stage (Fig. 4 and Supplementary Table S3).

**Fig. 5:**
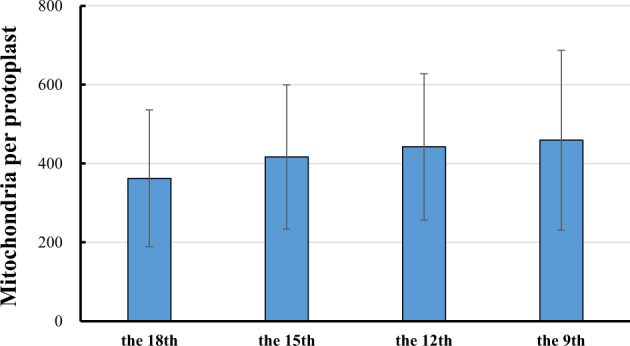
Number of mitochondria per cell at the four stages of melon leaf development

**Fig. 6:**
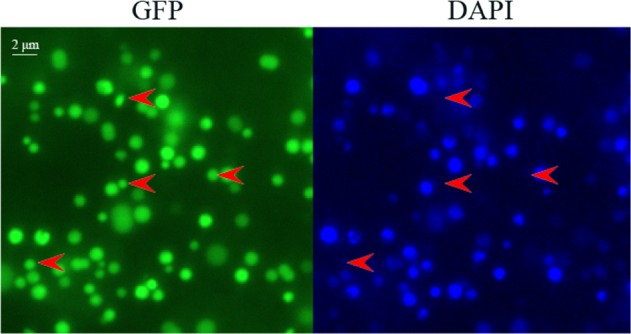
Mitochondria isolated from melon. Leaves from transgenic melon with mitochondrially targeted GFP (35S-coxIV-GFP) were stained with DAPI and visualized at excitation wavelengths for green fluorescent protein (left) and DAPI (right). It is clear that mtDNA signals were not present in a portion of the mitochondria. The red arrows highlight mitochondria without mtDNA signals. Bar = 2 μm

The mean numbers of mitochondria at the four stages (from youngest to oldest) were 362.1 ± 173.6, 416.6 ± 182.7, 442.2 ± 185.3, and 459.1 ± 228.2, respectively (Fig. 5). No significant differences in the number of mitochondria per cell were observed between the four stages due to large standard deviations, although on average, there was a trend towards increased numbers of mitochondria throughout leaf development. However, this trend was not consistent with the changes in mitochondrial gene copy numbers. Because leaf cells contained only 36.8–82.9 copies of the mitochondrial genome, these results imply that the majority of mitochondria do not contain the entire mitochondrial genome. In support of this observation, some mitochondria showed no detectable DAPI signals (Fig. 4). To further investigate DAPI signals in the mitochondria, these organelle were isolated from a mixture of melon leaves ranging from the 9th to 18th leaves, and mtDNA was not detected by DAPI staining in 4% of the mitochondria (*n* = 2000) (Fig. 6). This frequency was far below that reported for leaf cells of *Arabidopsis*^6^. In reality, most mitochondria likely have mtDNA, and its absence may have been due to the limited resolution of DAPI staining. Nevertheless, our results suggest that the mitochondrial genome of melon is split into small sub-genomes and that each mitochondrion may carry only parts of the mitochondrial genome.

## Discussion

### The copy number of mitochondrial genes does not correlate with the copy number of mitochondrial genomes

The copy numbers of four mitochondrial genes were investigated in melon leaf cells during development and were found to range from 36.8 (*atp6* copies in the 15th leaf, Fig. 2c) to approximately 82.9 (*nad9* copies in older 9th leaf, Fig. 2c). These numbers are lower than the estimated copy numbers of the mitochondrial genome in dark-grown shoots of several cucurbit species (110–140), as determined by reassociation kinetics^14^. However, these numbers are in agreement with the copy numbers of mitochondrial genomes previously reported for the leaves of *Arabidopsis*, barley and tobacco^6,9^. Compared with previous methods, ddPCR assays can determine the absolute copy number of nucleic acids in a sample without reference standards and unequal PCR efficiencies during amplification of individual genes, which provides increased precision and sensitivity at several orders of magnitude more than real-time PCR and even next-generation sequencing technologies^22,23^. Therefore, the copy numbers of the four mitochondrial genes could reflect the real copy numbers of the regions of the mitochondrial genomes among the different mitochondrial genes during leaf development.

Given the differences in gene copy numbers and the consistency for *nad9*, *rps1*, and *matR* before maturation of melon leaves, the four mitochondrial genes were located on at least two independent sub-genomic molecules, which is consistent with published assembly results^15^. However, even though *nad9* and *rps1* are located adjacently on one scaffold^15^, differences in their copy numbers were observed during melon leaf maturation. Therefore, the sub-genomic molecules of the mitochondrial genome may be dynamic, and these differences could be due to separation onto different mtDNA molecules. This idea indicates that the mitochondrial genome of melon does not exist as a single master chromosome and may explain why the mitochondrial genome of melon could not be assembled into one scaffold^15^. We hypothesize that the mitochondrial genome of melon exists as a sub-genomic molecule, such as that of cucumber and *Silene*^10,16^. The physical map may represent the predominant structure of the melon mitochondrial genome. The mitochondrial genes of melon are likely distributed across independent sub-genomic molecules, even for two genes located adjacently on the same scaffold (*nad9* and *rps1*). The copy numbers of these genes changed during leaf development, and differential amplification of sub-genomic molecules occurs, which likely produced different absolute values. Therefore, the copy numbers of mitochondrial genes were different, estimates of the copy numbers of one mitochondrial gene will not reflect the copy numbers of other mitochondrial genes in melon, and copy numbers of each gene need to be independently measured.

### The copy number of mitochondrial genes is less than the number of mitochondria

We observed that the copy numbers of mitochondrial genes are lower than the numbers of mitochondria, which is consistent with the findings from the leaf cells of *Arabidopsis*, tobacco, and barley^9^. Our data demonstrate that the leaf cells of melon do not contain sufficient copies of mitochondrial genes to ensure that every mitochondrion possesses the whole mitochondrial genome. Given that the vast majority of mitochondria contain DAPI signals, most or all the mitochondria of melon leaf cells may contain only parts of the mitochondrial genome. Again, the results obtained in this research are consistent with our conclusion by which the mitochondrial genome of melon exists as sub-genomic molecules.

The existence of sub-genomic molecules in the mtDNA of melon raises intriguing questions about the stability and transmission of mtDNA. Because mitochondria and mtDNA do not arise de novo, newly formed daughter cells must inherit mitochondria with the mitochondrial genome from parental gametes^24^. Melon undergoes paternal transmission of mtDNA^13^, and high levels of mtDNA (1296.3 ± 310.6 copies) were found in the generative cell of melon based on an analysis of one mitochondrial gene, matR^6^. Using the same mitochondrial gene, we demonstrated that the leaf cells of melon contain 44.5–78.6 copies of mtDNA. Therefore, the mtDNA levels of gametic cells are 16.5–29.1-fold (1296.3 vs. 44.5–78.6) greater than those of melon leaf cells. Although the results of Wang et al. were from an analysis of only one mitochondrial gene, cytological evidence confirmed the increase in mtDNA in the generative cell of melon^6^. Consistent with maternal transmission of mtDNA, the male gametic cells from *Arabidopsis*, *Antirrhinum majus*, and *Nicotiana tabacum* possess much less mtDNA, at 0.083, 0.47, and 1 copy on average, respectively^6,11^. These studies reveal that the copy number of mtDNA in gametes is regulated and consistent with mitochondrial transmission. The mtDNA of melon undergoes paternal transmission and therefore likely passes through a “bottleneck”, in which relatively few mitochondria are transferred via sperm cells to the progeny, as described in the female germline in mammals^25,26^. Because mtDNA is transmitted within the mitochondrion, it is likely that during development of the sporophyte and gametophyte, mitochondria undergo fusion and allow recombination among direct repeats on the sub-genomic molecules, which is an irreversible process^27^. In cucumber, the three mitochondrial chromosomes exhibit striking differences in their recombinational dynamics^16^. Our previous studies demonstrated that the mitochondrial genome of cucumber does not exist solely as these three chromosomes and is more structurally complex due to recombination^28^. The rearranged mtDNAs can sort among mitochondria^28^ and can be transmitted to progeny, allowing the selection of superior recombinant molecules to maintain the integrity of the mitochondrial genome.

Genes remaining in mtDNA are critical for mitochondrial function, and thus, the entire collection of genes is presumably required in each mitochondrion to ensure the integrity of function^29^. Comprehensive analysis of mtDNA content clearly indicates that every mitochondrion may possess parts of mtDNA and potentially mtDNA in leaf cells at such low levels not detectable by ddPCR. Therefore, frequent fusion and fission of mitochondria could allow for the combination and exchange of genetic information^29,30^. However, the biological significance of the division of mtDNA into sub-genomes and how this benefits cells should be the focus of future research.

## Materials and methods

### Plant materials

Seeds of a doubled haploid (DHL92) line of melon were kindly provided by Jordi Garcia-Mas of the Centre for Research in Agricultural Genomics (CSIC-IRTA-UAB-UB, Barcelona, Spain). The seeds were germinated in “Type-P” soil (Hawita Gruppe GmbH, Vechta, Germany) in the dark at 28 °C in April, after which the seedlings were grown under 12 h light (28 °C)/12 h dark (25 °C) conditions until the expansion of the first true leaf. The plants were then transplanted to plastic houses at Yangdu, Jiaxing, China, in the spring of 2017 and grown under greenhouse conditions.

### Paraffin sections

The sizes of leaf cells were measured by paraffin sections stained with safranin-fast green. The 9th, 12th, 15th, and 18th leaves were harvested and immediately fixed in formalin/glacial acetic acid (FAA). The samples were then dehydrated in a xylene and alcohol series (75, 85, 90, 95 and 100%) and then embedded in paraffin wax. Four-micrometer-thick sections were cut and stained with safranin (1%)-fast green (0.5%) for histological examination. The lengths and widths of the leaf cells (*n* = 30 for each stage) were measured with CaseViewer 2.1 (3DHISTECH, Budapest, Hungary).

### Flow cytometric analysis

Flow cytometric measurements of nuclei suspensions from fresh leaves were performed as described by Barow and Meister using a FACSAria flow cytometer (BD Biosciences, San Jose, CA, USA)^17^. The ploidies of 10000 nuclei from each sample were measured and analyzed with Cell Quest 3.3 (BD Biosciences). The C level, which reflects the DNA content in terms of multiples of the monoploid (C) genome, was calculated as a weighted average using the formula [(2*n_2C_) + (4*n_4C_) + (8*n_8C_) + (16*n_16C_) + …]/(*n*_2C_ + *n*_4C_ + *n*_8C_ + *n*_16C_ + …), where “*n*” is the number of nuclei whose DNA content corresponds to 2C, 4C, 8C, or 16C^9^.

### Evaluation of mtDNA copy numbers by ddPCR

Droplet digital PCR (ddPCR) was performed to evaluate the copy numbers of mitochondrial genes^22,31^. The ddPCR system included an automated droplet generator and reader (QX200 Droplet Digital PCR, Bio-Rad, Hercules, CA, USA). For mtDNA quantification, four sets of primers and probes targeting the sequences of mitochondrially encoded *nad9*, *rps1*, *matR*, and *atp6*, respectively^15^, and two sets of primers and probes targeting the sequences of nuclear DNA (nDNA) encoding *CmDPD1* (MELO3C015276) and *CmWhy2* (MELO3C022122.2) were selected for the quantification of their absolute copy numbers by ddPCR. All primers and probes (Supplementary Table S1) used were designed by Primer Express 3.0.1. To avoid nuclear mtDNA sequences (NUMTs), we performed BLAST searches flanking each amplicon against the melon genome^32^ (https://www.melonomics.net/melonomics.html), and the primers and probes with neither hits nor complete alignment with nuclear sequences were selected for ddPCR. Probes targeting mtDNA were labeled with FAM fluorophore, whereas the probe targeting nDNA was labeled with HEX. All probes had the BHQ1 quencher. The ddPCR method was performed according to the manufacturer’s instructions modified by the use of melon genomic DNA and different temperatures for annealing/extension during PCR. The DNA samples were fractionated into ~20000 droplets. Serial dilutions were performed, after which 50 ng of DNA was loaded into the final reactions; 56 °C was used for the annealing/extension in the final PCR. The data were analyzed using QuantaSoft software (Bio-Rad, Hercules, CA, USA), which determines the numbers of droplets that were positive and negative for each fluorophore in each sample. The fraction of positive droplets was then fitted to a Poisson distribution in QuantaSoft to determine the absolute copy number per microliter.

### Nucleic acid extraction and quantitative reverse transcription (RT)-PCR

Total DNA and RNA were isolated from melon leaves at different developmental stages using the DNeasy Plant Mini Kit and RNeasy Plant Mini Kit (Qiagen, Hilden, Germany) according to the manufacturer’s protocols, respectively. The qualities of DNA and RNA were checked by 1% agarose gels. Concentrations of the DNA and RNA were determined spectrophotometrically using a NanoDrop 2000 system. cDNA was synthesized from 1000 ng of DNase I-treated (Qiagen) total RNA after priming with oligo (dT) in a 20-mL reaction via the EasyScript cDNA Synthesis Kit (Lamda Biotech, St. Louis, MO, USA). For qRT-PCR analyses, the mitochondrial genes *nad9*, *rps1*, *matR*, and *atp6* were chosen, and the nuclear housekeeping gene *Actin* (MELO3C008032) was used to normalize the qPCR output. The primers used (Supplementary Table S1) were designed according to the melon mitochondrial and nuclear gene sequences^15,32^. Amplification was conducted using Maxima SYBR Green qPCR Master Mix (Thermo Fisher Scientific) and StepOne (Thermo Fisher Scientific), with 40 cycles of denaturation at 94 °C for 30 s, annealing at 60 °C for 30 s and extension at 72 °C for 30 s. The amplification specificity was tested by a dissociation curve (65–90 °C) to compare the results from different reactions and samples. The expression levels of mitochondrial genes were normalized to the value for *Actin*. At least three independent biological and technical replicates were tested per sample. The primer efficiencies were estimated with a dilution series, and the relative gene expression levels were determined by 2^−△△Ct^ formula^33^.

### Transformation of melon

Transgenic melon expressing a mitochondrially targeted GFP (35S-coxIV-GFP)^34^ was developed. The fragment carrying 35S-coxIV-GFP was amplified from the transgenic cucumber line “B” using specific primers^12^ and inserted into the expression vector pCAMBIA2301 between the *Hind*III and *Sac*I sites. The plasmid was subsequently transformed into *Agrobacterium tumefaciens* strain LBA4404 by the freeze–thaw method, which was then transformed into melon cultivar “S7” using cotyledon explants as described previously^35^. T_0_ plants with the integrated transgene were confirmed by PCR and fluorescence microscopy.

### Protoplast isolation and determination of the number of mitochondria

Protoplasts were isolated from melon leaves using the method described by Yoo et al.^36^ Briefly, the leaves were cut into 0.5–1 -mm strips using a new, sharp razor blade, and the strips were transferred carefully into an enzyme solution (1.5% cellulase R10, 0.4% macerozyme R10, 0.4 M mannitol, 20 mM KCl, 10 mM CaCl_2_, 0.1% BSA, 1 mM β-mercaptoethanol, and 20 mM MES, pH 5.7). A vacuum was then applied to infiltrate the leaf strips for 30 min in the dark using a desiccator, after which the strips were gently shaken in the dark for at least 3 h at room temperature. The protoplasts were washed and filtered twice with W5 solution (154 mM NaCl, 125 mM CaCl_2_, 5 mM KCl and 2 mM MES, pH 5.7) and a 75-μm nylon mesh, after which they were centrifuged at 100 *g* for 1 min. The protoplast pellets were resuspended in W5 solution and incubated on ice for 30 min. The protoplasts were finally resuspended in MMG solution (0.4 M mannitol, 15 mM MgCl_2_, and 4 mM MES, pH 5.7) and kept on ice.

For counting mitochondria, fresh protoplasts isolated from leaves were transferred to a microscope slide, stained with 5 mM DAPI in TAN buffer (20 mM Tris-HCl, 0.5 mM EDTA, 7 mM b-mercaptoethanol, and 1.2 mM spermidine, pH 7.6), and gently squashed under a cover slide as described previously^37^. The samples were then incubated for 2–3 min at room temperature in the dark and then observed with an Olympus BX51 fluorescence microscope. The charged-coupled device (CCD) camera was controlled by FISH View 5.5 software (Applied Spectral Imaging, Carlsbad, CA, USA), and the images were processed by Adobe Photoshop CS4. The number of mitochondria per protoplast were determined using the “Analyze Particles” function of the software program ImageJ (https://imagej.nih.gov/ij/download.html). Mitochondria in 30 randomly chosen protoplasts at each developmental stage were counted.

### Isolation of mitochondria from melon leaves

Mitochondria were isolated from melon leaves by differential centrifugation through Percoll-density gradients^38^. Briefly, melon leaves ranging from the 18th to 9th leaf were used as samples, and everything including buffers and the equipment used was at 4 °C. The leaves were chopped in a blender in 400 mL of homogenization buffer [400 mM mannitol, 1 mM EGTA, 25 mM MOPS–KOH, 10 mM tricine, 8 mM cysteine (added the day of the extraction), 0.2% (w/v) BSA, and 1% (w/v) PVP-40, pH 7.8] for 15 s at low setting three times. The homogenized tissue was then filtered through eight sheets of cheesecloth and two sheets of Miracloth, and the filtrate was subsequently centrifuged at 1000 × *g* for 10 min to remove any cellular debris. The supernatant was then centrifuged at 3000 × *g* for 10 min to remove the nuclei. The resulting supernatant was centrifuged at 17000 × *g* for 20 min to pellet the mitochondria. The pellet was resuspended in the homogenizing buffer and transferred to the Percoll gradient slowly, which was centrifuged at 18000 × *g* for 60 min. The white band of mitochondria was extracted from the 33–80% interface of the Percoll and washed with 15 mL of washing buffer twice by centrifugation at 18000 × *g* for 20 min each time. The mitochondria were stained with DAPI and then visualized under a fluorescence microscope.

## Disclaimer

Names are necessary to report factually on available data; however, the U.S. Department of Agriculture (USDA) neither guarantees nor warrants the standard of the product, and the use of the name by the USDA implies no approval of the product to the exclusion of others that may also be suitable.

## Supplementary information

Supplementary Tables
