# Supplementary material for: Copy numbers of mitochondrial genes change during melon leaf development and are lower than the numbers of mitochondria
Source: Hortic Res. 2019 Aug 11;6:95. doi: 10.1038/s41438-019-0177-8 (PMC6804604; doi:10.1038/s41438-019-0177-8)
Supplement: Supplementary file 1 — Supplementary Tables [file 41438_2019_177_MOESM1_ESM.docx]

**Supplementary Table S1. Primers used in this study.**

| **Name** | **Forward primer (5’ to 3’)** | **Reverse primer (5’ to 3’)** |
| --- | --- | --- |
| **Primer for *nad9*** | CGGATGATTGATGGAAGAAACA | CAGTGCAGACGAAGTAACACGAA |
| **Probe for *nad9*** | (FAM)-CAAACATATCCCAAACTTCTCGCTCCCAC-(BHQ1) |  |
| **Primer for *rps1*** | GCCTTATGAGAGGACAGAATGGA | GGATGGAACTGAACAAGATTTGG |
| **Probe for *rps1*** | (FAM)-ATGAAACCCGCGATGGCTACTGAATAACC-(BHQ1) |  |
| **Primer for *matR*** | GTAAGCAGGCCCCCTTAAAA | TAGGAAGTGGGCGAGACGTT |
| **Probe for *matR*** | (FAM)-CGCGATATGCCGACGACTTACTACT-(BHQ1) |  |
| **Primer for *atp6*** | TTCAGAGTTCGTCCGCAACAT | CTAGTACCGGGCAAGGTTCTCA |
| **Probe for *atp6*** | (FAM)-TAACTTCTAGCTTCCGATTCCCCTTCCCC-(BHQ1) |  |
| **Primer for *CmDPD1*** | GCTCATAATGCTCGTACCTTCGA | ACTTCATCAGTTGACGGGCTAAC |
| **Probe for *CmDPD1*** | (HEX)-CCCTTTTTACTCCACGAATTCAGCCGTT-(BHQ1) |  |
| **Primer for *CmWhy2*** | TGAATGTGTATCATAGTCTGGGAATCT | GTAACCTGTTTCCTTGTCCAATCAT |
| **Probe for *CmWhy2*** | (HEX)-TGGTTCAATAATGCTGACATTCGCTCCTG-(BHQ1) |  |
| **Probe2 for *CmWhy2*** | (FAM)-TGGTTCAATAATGCTGACATTCGCTCCTG-(BHQ1) |  |
| ***Actin*** | ATTCTTGCATCTCTAAGTACCTTCC | CCAACTAAAGGGAAATAACTCACC |
| **Cloning of 35S-coxIV-GFP** | GGTTCGCTTGCTGTCCATAA | CGGCAACAGGATTCAATCTTAA |
| **PCR for transgenic melon** | TTGATGTGATGGTCCGATTGAG | GTCCTTGAAGAAGATGGTCCTC |

Note: Underlines are the fluorophores and quenchers designed in primers.

Supplementary Table S2. The effect of the two endogenous genes on the assessment of reference gene copy numbers.

| Sample | *CmDPD1* Conc (copies/μL) | *CmWhy2* Conc (copies/μL) |
| --- | --- | --- |
| the 18th | 33.2 ± 2.3 | 34.6 ± 0.8 |
| the 15th | 36.5 ± 1.0 | 34.9 ± 1.3 |
| the 12th | 45.8 ± 2.6 | 46.2 ± 3.1 |
| the 9th | 56.2 ± 3.7 | 55.5 ± 3.2 |

The probe targeting *CmDPD1* was labeled with HEX fluorophore, and the probe targeting *CmWhy2* was labeled with FAM fluorophore. 50 ng DNA was used for ddPCR. Duplex ddPCR (mixing of primers and probes in the same reaction) was used to generate droplets at the same time.

Supplementary Table S3. The number of mitochondria per protoplast during leaf development of melon.

| Sample | the 18th | the 15th | the 12th | the 9th |
| --- | --- | --- | --- | --- |
| 1 | 223 | 578 | 391 | 442 |
| 2 | 511 | 496 | 476 | 578 |
| 3 | 312 | 358 | 373 | 512 |
| 4 | 346 | 241 | 962 | 325 |
| 5 | 358 | 378 | 671 | 336 |
| 6 | 214 | 149 | 395 | 936 |
| 7 | 408 | 296 | 299 | 912 |
| 8 | 578 | 387 | 385 | 996 |
| 9 | 148 | 345 | 241 | 338 |
| 10 | 125 | 578 | 352 | 169 |
| 11 | 436 | 664 | 126 | 99 |
| 12 | 347 | 589 | 578 | 667 |
| 13 | 98 | 365 | 398 | 496 |
| 14 | 169 | 396 | 649 | 551 |
| 15 | 432 | 478 | 274 | 332 |
| 16 | 396 | 598 | 527 | 198 |
| 17 | 91 | 148 | 368 | 226 |
| 18 | 86 | 896 | 678 | 554 |
| 19 | 546 | 674 | 438 | 287 |
| 20 | 397 | 345 | 346 | 289 |
| 21 | 472 | 264 | 223 | 396 |
| 22 | 312 | 299 | 467 | 446 |
| 23 | 399 | 678 | 110 | 664 |
| 24 | 385 | 168 | 556 | 521 |
| 25 | 369 | 285 | 419 | 698 |
| 26 | 568 | 365 | 337 | 378 |
| 27 | 742 | 328 | 544 | 466 |
| 28 | 197 | 612 | 691 | 96 |
| 29 | 663 | 298 | 668 | 388 |
| 30 | 542 | 243 | 325 | 478 |
| Average | 362.3 | 416.6 | 442.2 | 459.1 |
| Standard deviation | 173.6 | 182.7 | 185.3 | 228.2 |
